# Organizational support, training and equipment are key determinants of burnout among dialysis healthcare professionals during the COVID-19 pandemic

**DOI:** 10.1007/s40620-022-01418-6

**Published:** 2022-08-30

**Authors:** Ewa Pawłowicz-Szlarska, Joanna Forycka, Karolina Harendarz, Martyna Stanisławska, Agnieszka Makówka, Michał Nowicki

**Affiliations:** 1 Department of Nephrology, Hypertension and Kidney Transplantation, Medical University of Lodz, Pomorska Str. 251, 91-213 Lodz, Poland; 2 Student Scientific Society affiliated with the Department of Nephrology, Hypertension and Kidney Transplantation, Medical University of Lodz, Lodz, Poland

**Keywords:** Burnout, COVID-19, Dialysis units, Pandemic perceptions

## Abstract

**Introduction:**

Burnout was already found to be an important factor in the professional landscape of nephrology prior to the COVID-19 outbreak and is expected to worsen during the pandemic.

**Objectives:**

The aim of our study was to assess pandemic experiences, perceptions, and burnout among Polish dialysis unit professionals in the COVID-19 period.

**Participants and methods:**

A survey, which consisted of a Pandemic Experiences and Perceptions Survey (PEPS) and a Maslach Burnout Inventory was distributed online to Polish dialysis units. The study group comprised 379 participants (215 nurses, 148 physicians, and 16 respondents of other professions).

**Results:**

The pandemic largely affected or completely dominated the work of dialysis units according to 53.4% and 25.5% of nurses responding to the PEPS, respectively. Among physicians, the prevalence was 55.5% and 15.4% of participants, respectively. Serious or life-threatening risk was perceived by 72.1% and 11.9% of dialysis healthcare professionals, respectively. Furthermore, 74.6% of the study participants stated that their work in a dialysis setting amidst the pandemic was felt to be associated with serious risk for their relatives. Adequate personal protective equipment and information from management decreased burnout among dialysis staff. Burnout was lower in all dimensions among those participants who felt more in control of their exposure to infection, provided by proper training, equipment, and support (*p* = 0.0004 for emotional exhaustion, *p* = 0.0007 for depersonalization, and *p* < 0.0001 for feelings of personal accomplishment).

**Conclusions:**

The COVID-19 pandemic has largely affected the work in dialysis units. Providing proper training, equipment, and support may decrease burnout among dialysis staff.

**Graphical abstract:**

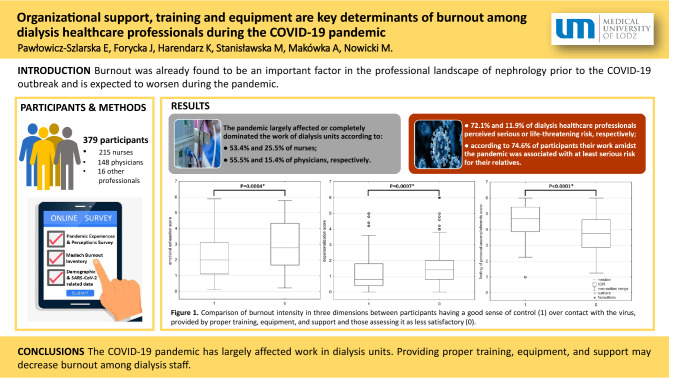

## Introduction

The prolonging current COVID-19 pandemic has been caused by a novel coronavirus (SARS-CoV-2), which first appeared in Wuhan in November 2019 and rapidly spread globally. The World Health Organization declared a Public Health Emergency of International Concern on 30 January, 2020 and a pandemic on 11 March, 2020 [1, 2]. After two years, as of 11 April, 2022, there were 497,057,239 confirmed cases of COVID-19 including 6,179,104 deaths worldwide [3]. According to the Polish Registry of Renal Replacement Therapy, 5271 cases of COVID-19 were recorded among dialysis patients in 2020 (21% of the dialysis population), including 5187 among hemodialysis (HD) patients and 84 cases among peritoneal dialysis patients. Twenty-eight percent of infected hemodialysis patients died within 6 weeks of diagnosis [4]. In 2021, 4129 cases of COVID-19 were recorded among Polish dialysis patients (16% of the whole dialysis population) [5].

The COVID-19 pandemic is an unprecedented challenge for healthcare systems around the world, leading to staff shortages that limit treatment options for patients without COVID-19 [6], and forcing organizational changes, i.e., introducing telemedicine into everyday practice [7]. The impact of COVID-19 on the well-being, psychological distress, and burnout of healthcare professionals has been addressed in numerous studies [8–12].

The meaning of mass disasters for burnout in nephrology personnel is discussed in detail by Sever et al. During and after a catastrophe, healthcare providers may be affected by a number of infrastructural, organizational, and emotional problems as well as increased workload. The factors that may influence burnout after a mass disaster (such as the COVID-19 pandemic) comprise high numbers of patients, increased healthcare demands, suboptimal resources, and conditions leading to insufficient care being provided to patients, thus giving rise to a feeling of failure and safety concerns associated with insufficient personal protection equipment.

Specific dialysis-related factors associated with the COVID-19 pandemic influencing the well-being of healthcare professionals comprise outbreaks in hemodialysis units, high numbers of additional patients with acute kidney injury due to COVID-19 [13] as well as poor COVID-19 outcomes of maintenance HD patients with mortality rates exceeding 20% [14]. As a life-saving procedure, HD could not undergo deep organizational adjustments, which was the case for other out-patient facilities that largely turned towards telemedicine services.

Burnout was already found to be an important factor in the professional landscape of nephrology prior to the COVID-19 outbreak. Among the unique characteristics of renal care settings that may contribute to burnout, we must highlight the use of technologically advanced equipment, the intensive care environment, and the long-term relationships that are established between the healthcare professionals and chronic renal patients [15]. Other specific risk factors comprise the high complexity of kidney patients [16] and reductions in the nephrology workforce leading to work overload [17]. As we reported in our previous study on burnout among Polish nephrologists, physicians working mostly in dialysis settings might be at increased risk of reduced personal accomplishment compared to their colleagues working in in-patient nephrology departments [18].

Burnout among nephrologists has been an increasingly discussed topic in recent years, which indicates a growing understanding of its relevance for both patient satisfaction [19] and the nephrology workforce [20]. The influence of the current pandemic on burnout among renal healthcare professionals has so far only been addressed in two reports—the international survey on COVID-19 experiences which enrolled mostly nurses [21] and the national UK report on the influence of long COVID-19 on burnout and work life among the nephrology workforce [22].

The aim of our study was to evaluate pandemic experiences, perceptions, and burnout among Polish doctors and nurses working in dialysis centers in the COVID-19 era. Since the current experiences may influence the staff’s psychological condition, motivation, and future career decisions, it is crucial to ensure accurate monitoring of burnout burdens thus enabling subsequent preventive actions.

## Participants and methods

### Study survey

An online survey hosted on the *Momentive* application was distributed via e-mail and social media platforms. The survey, the study protocol, and the opinion of the local ethics committee were first sent to the Polish National Consultant in Nephrology, the President of the Polish Society of Nephrology, and the President of the Polish Society of Nephrology Nurses with the request to endorse and support the project. Upon endorsement and approval received from these officials, the survey was sent to Regional Consultants in Nephrology, with a request to spread the survey in their regions, to the societies’ members via e-mail and posted on the Facebook profiles of the Polish Society of Nephrology and the Polish Society of Nephrology Nurses.

The survey was open from 1st September, 2021 to 31st December, 2021. The approximate time to complete the survey was estimated to be 12–15 min.

The first page of the survey contained an introductory letter stating that completing the questionnaire is tantamount to giving informed consent to participate in the project. The letter stated also that the survey was completely anonymous and the rationale for the project was briefly described. Expressing consent made it possible to move on to the next questions of the survey.

The study survey tools consisted of two psychometric instruments—Maslach Burnout Inventory—Human Services Survey for Medical Personnel—MBI-HSS (MP) and Pandemic Experiences and Perceptions Survey (PEPS), as well as self-created questions. The instruments were used in accordance with the license agreements gained from MindGarden, Inc (www.mindgarden.com).

The Maslach Burnout Inventory (MBI) created by Christina Maslach and Susan E. Jackson is recognized worldwide as a gold standard for burnout assessment and was validated by extensive research [23].

MBI—Human Services Survey for Medical Personnel is derived from the Human Services Survey specifically for healthcare practitioners. The 22-item MBI-HSS (MP) addresses three dimensions of burnout:

- emotional exhaustion addressing feelings of being emotionally overextended and exhausted by one's work (9 items),
- depersonalization addressing an unfeeling and impersonal response toward patients (5 items),
- personal accomplishment addressing feelings of competence and successful achievement in one's work (8 items).

Every statement in the MBI-HSS (MP) was assessed on the time scale (never—0 points, a few times a year—1 point, once a month or less—2 points, a few times a month—3 points, once a week—4 points, a few times a week—5 points and every day—6 points).

Adding the points of particular items allowed qualifying burnout in all three dimensions as low, moderate, and high. It is important to note that higher scores in emotional exhaustion and depersonalization reflect greater burnout, while higher scores in personal accomplishments mean lower burnout in this dimension. The scores for the particular dimensions were as follows: personal accomplishment: ≥ 40 low, 34–39 moderate, < 34 high burnout; depersonalization: < 6 low, 6–9 moderate, ≥ 10 high burnout; emotional exhaustion: < 19 low, 19–26 moderate, ≥ 27 high burnout.

The Pandemic Experiences & Perceptions Survey (PEPS) by Michael P. Leiter is a tool used to measure the experiences of employees working during a pandemic. Such an assessment may provide leaders with key guidance for managing the current situation, leading the organizational recovery afterward, and for anticipating future challenges. The PEPS assessment provides critical information on the extent of workflow disruption, resource adequacy, risk perception, impact on work life, and perceptions of leadership [24].

Participants were also asked about the specific burnout risk factors which were identified during interviews with ten experienced dialysis professionals. This strategy was applied to minimize author bias. The following factors were identified: (1) repeated complaints from patients, (2) lack of professional challenges, (3) no significant progress in dialysis care in recent years, (4) high mortality rate in the dialysis population, (5) nonadherence among dialysis patients, (7) chronic character of treatment, and (8) no causal treatment for most of the patients.

The demographic and work-related data collected as part of the survey included gender, age, time of professional experience, profession, and main workplace. The history of SARS-CoV-2 infection among respondents and their close relatives was also addressed. All questions, except for informed consent, were answered voluntarily. The study protocol was approved by the local ethics committee of the Medical University of Lodz.

### Study group

Physicians, nurses, and other professionals (i.e., technicians, administration specialists) working in dialysis units in Poland were eligible to complete the survey. Inclusion criteria were as follows: (1) employment in a dialysis unit and (2) consent to participate in the study as described above. Out of 392 respondents who opened the link with the survey, 379 gave their consent (96.7%). The study group characteristics are provided in Table 1. Due to the small number of other healthcare professionals (*N* = 16), all comparisons presented in the study were limited to physicians (*N* = 148, 39.1%) and nurses (*N* = 215, 56.7%).

**Table 1:** The study group characteristics (data presented as N (%), mean ± standard deviation (SD) or median [interquartile range (IQR)])

Characteristic	Physicians (*N* = 148)	Nurses (*N* = 215)	Other professionals (*N* = 16)
Gender [*N*, (%)]			
Males	70 (47.3%)	5 (2.3%)	7 (43.8%)
Females	77 (52%)	208 (96.8%)	8 (50%)
Other	1 (0.7%)	0 (0%)	1 (6.2%)
No data	0 (0%)	2 (0.9%)	0 (0%)
Age (years) [mean ± SD]	50.2 ± 10.4	47.9 ± 9.3	36.5 ± 9.2
Work experience in dialysis facilities (years) [median [IQR]]	18 [10–26]	21 [11–28]	4.5 [3–14.5]
The share of work in a dialysis center in relation to the total employment			
The only workplace	23 (15.6%)	171 (79.5%)	11 (68.8%)
Main workplace	105 (70.9%)	38 (17.7%)	4 (25%)
Additional workplace	20 (13.5%)	6 (2.8%)	1 (6.2%)
Occupation subgroups	Nephrologist 134 (90.5%)	–	Technician 8 (50%)
	Internist 10 (6.8%)		Administration specialist 8 (50%)
	Resident 4 (2.7%)		
SARS-CoV-2 infection [*N*, (%)]	58 (39.2%)	70 (32.6%)	5 (31.2%)
Symptomatic	52 (89.7%)	61 (87.1%)	5 (100%)
Mild Symptoms	11 (21.1%)	12 (19.7%)	1 (20%)
Moderate	25 (48.1%)	18 (29.5%)	2 (40%)
Severe (home)	16 (30.8%)	27 (44.3%)	2 (40%)
Hospitalization	0 (0%)	4 (6.5%)	0 (0%)
COVID-19 among relatives	84 (56.8%)	123 (57.2%)	12 (75%)
Death due to COVID-19 among relatives	7 (4.7%)	20 (9.3%)	1 (6.3%)

### Statistical analysis

Results are presented as mean ± standard deviation (SD) or median and interquartile range (IQR) depending on the normality of the distribution of each variable assessed with the use of the Shapiro–Wilk test. Statistical analysis was performed using Statistica ver. 13.1 PL software. Graphs were plotted with MS Excel and Statistica. Mann–Whitney *U* test was used for nonparametric comparisons between two independent groups. The Chi-square tests were used for comparisons of categorical data. Correlations were assessed with rank-order Spearman’s method. Pairwise deletion of missing data was applied.

## Results

### Burnout assessment

**Fig. 1:**
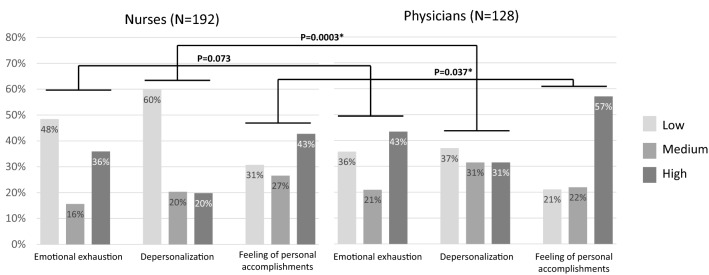
Percentage of low, moderate and high burnout levels in particular dimensions and comparison of prevalence patterns among nurses and physicians (chi-square Pearson test, statistically significant *p* values are marked with asterisk)

According to the Maslach Burnout Inventory, emotional exhaustion did not differ between nurses and physicians, while depersonalization was significantly lower and feelings of personal accomplishment significantly higher among nurses than among physicians. Detailed results of the survey are provided in Table 2. The burnout level in three dimensions perceived by dialysis nurses and physicians during the COVID-19 pandemic is presented in Fig. 1. Three-dimensional burnout was found among 15% (*N* = 48) of participants (high burnout levels in all dimensions).

**Table 2:** Comparison of burnout mean scores in all dimensions between nurses and physicians (data presented as median and interquartile range (IQR), Mann–Whitney *U* test, statistically significant *p* values are marked with asterisk)

Burnout dimension	Nurses (*N* = 192)	Physicians (*N* = 128)	*p* value
Emotional exhaustion	2.1 (2.2)	2.7 (2.8)	0.052
Depersonalization	1 (1.2)	1.4 (1.4)	< 0.001*
Feeling of personal accomplishments	4.4 (1.8)	4 (1.4)	0.035*

Among nurses, a positive weak correlation (Spearman rank-order correlation; *r* = 0.25, *p* = 0.0005) between the feelings of personal accomplishment score and age was revealed, whereas no correlations between other dimensions scores and age were found. No associations between burnout scores and main workplace were found in this group. The occurrence of SARS-CoV-2 infection in the participant and/or their family members did not influence burnout scores.

Among physicians, neither gender nor age influenced burnout measures. Similarly, no associations between burnout scores and main workplace were found. The occurrence of SARS-CoV-2 infection in the participant and/or their family members did not influence burnout scores in the group of physicians either.

In the self-assessment part, 44.2% of nurses and 49.2% of physicians stated that they felt burned out. Among them, 85.7% of nurses and 76.2% of physicians confirmed that the feeling of burnout increased during the COVID-19 pandemic.

**Fig. 2:**
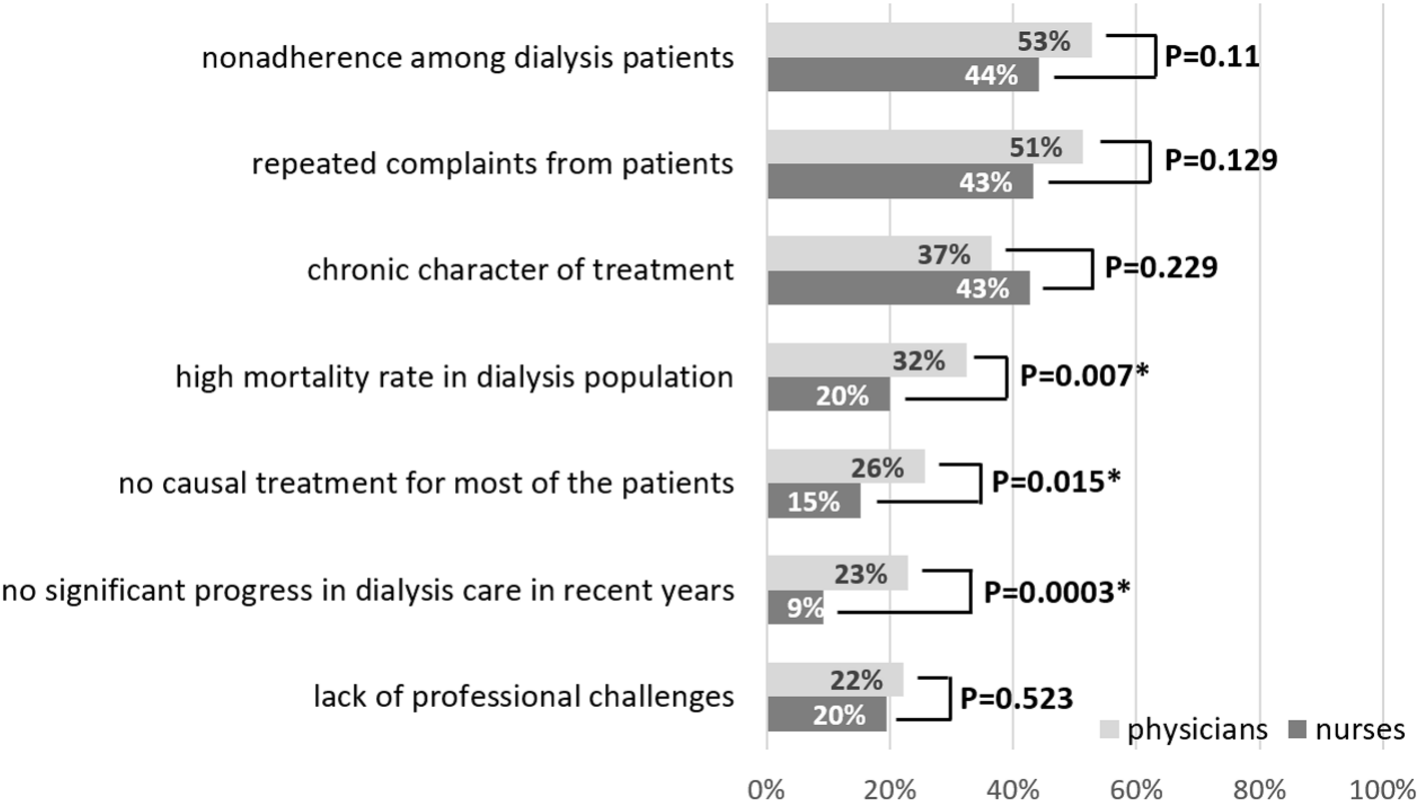
Factors which may increase burnout related to work in dialysis units comparing to work in other kidney care settings according to study participants (chi-square Pearson test, statistically significant *p* values are marked with asterisk)

Eighty percent of nurses and 84.4% of physicians stated that working in dialysis units is associated with specific risk factors, which may increase burnout compared to working in other kidney care settings. These factors, chosen by the participants from the preliminarily created list, are provided in Fig. 2.

The other factors enumerated most frequently by participants included: ‘monotony of work’, ‘constant contact with the same patients’, ‘lack of psychological support for dialysis patients who often “blame” staff for being sick’, ‘patients presenting reluctance to change habits and lifestyle’, ‘emotional attachment to patients due to the long-term nature of the therapy’.

### Pandemic experiences and perceptions and their associations with burnout

The pandemic largely affected or completely dominated the work of dialysis units according to 53.4% and 25.5% of nurses, respectively, while among physicians, rates were 55.5% and 15.4%, respectively. The assessment of the adequacy of personal protective equipment (PPE) and information from management as well as the sense of control over contact with the virus provided by training, equipment, and support is shown in Table 3.

**Table 3:** Nurses’ and physicians’ evaluation of work environment aspects at the time of the pandemic (data presented as *N* (%), Mann–Whitney *U* test, statistically significant *p* values are marked with asterisk)

Work environment characteristics at the time of the pandemic	Nurses	Physicians	*p* value
Adequacy of personal protective equipment (e.g. masks, gloves, etc.)	*N* = 160	*N* = 117	
Completely inadequate	5 (3.1%)	3 (2.6%)	
Barely adequate	9 (5.6%)	14 (8.8%)	
Somewhat adequate	28 (17.5%)	22 (13.7%)	0.314
Mostly adequate	36 (22.5%)	39 (24.4%)	
Completely adequate	82 (51.3%)	39 (33.3%)	
Adequacy of information from management	*N* = 159	*N* = 116	
Completely inadequate	8 (5.0%)	1 (0.8%)	
Barely adequate	9 (5.6%)	4 (2.5%)	
Somewhat adequate	27 (16.9%)	23 (19.7%)	0.617
Mostly adequate	37 (23.1%)	47 (40.2%)	
Completely adequate	78 (48.8%)	41 (35%)	
Sense of control over contact with the virus	*N* = 160	*N* = 117	
No control at all	7 (4.4%)	4 (3.4%)	
Minimal control	13 (8.1%)	12 (10.3%)	
Some control	50 (31.3%)	51 (43.6%)	0.064
A lot of control	76 (47.5%)	44 (37.6%)	
Complete control	14 (8.8%)	6 (5.1%)	

**Fig. 3:**
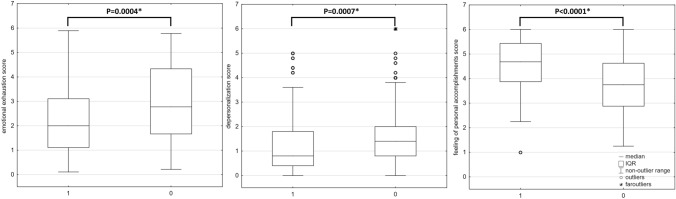
Comparison of burnout intensity in three dimensions between participants having a good sense of control over contact with the virus (1) and those assessing it as less satisfactory (0) (IQR—interquartile range, Mann–Whitney *U* test, statistically significant *p* values are marked with asterisk)

A comparison of burnout intensity in all dimensions between participants who assessed the adequacy of PPE positively (‘mostly adequate’ and ‘completely adequate’) and those who were not completely satisfied revealed higher scores for emotional exhaustion (*p* = 0.000334) and lower feelings of personal accomplishment (*p* = 0.026) in participants assessing PPE adequacy at most as ‘somewhat adequate’; no significant difference was found in terms of depersonalization (*p* = 0.083). A similar comparison performed for the adequacy of information showed a significant difference only for emotional exhaustion—those who rated information adequacy at most as ‘somewhat adequate’ scored higher in emotional exhaustion (*p* = 0.032). Significant associations were also found between burnout measures and a sense of control (‘a lot of control’ and ‘complete control’) over contact with the virus provided by proper support, training, and equipment (Fig. 3).

Nineteen point six percent of participants declared that they had direct daily contact with the virus during the pandemic, while 48.9% of them often had contact with the virus directly. As for the risk perception during the time of the pandemic, serious or life-threatening risk for the participant personally was perceived by 72.1% and 11.9% of dialysis healthcare professionals, respectively. Interestingly, 64.9% and 9.7% of the study participants stated that their work in a dialysis setting at the time of the pandemic was associated with serious or life-threatening risk for their relatives, respectively.

Management’s leadership skills, such as expressing hope for success, identifying actions that would improve capability, expressing confidence in staff capacity to take effective action, creating a sense of security, and honest assessment of the situation were all evaluated both by nurses and physicians with a median of 4 points on the 5-point Likert scale.

**Fig. 4:**
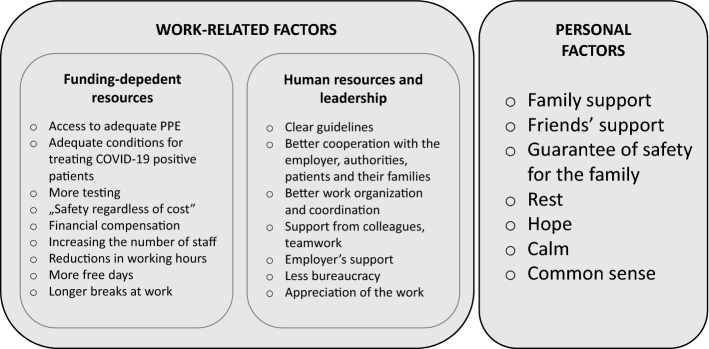
Three-dimensional framework of relieving factors at the time of pandemic

Analysis of factors that could help and might relieve staff during the pandemic period, enabled the creation of a three-dimensional framework (Fig. 4) of work-related and personal factors. Among factors that gave hope at the time of the pandemic, participants enumerated most often support from the family, faith, and belief in a quick end to the pandemic due to the introduction of vaccines.

## Discussion

Many studies published over the past two years have confirmed that the COVID-19 epidemic has significantly reduced the quality of life of healthcare professionals, aggravating pre-existing issues like burnout. As confirmed in a recent meta-analysis of 76 studies comprising 51,152 healthcare professionals from five continents, burnout levels increased from medium–high to high and compassion fatigue from medium to high [25]. In our study, we observed a high percentage of participants displaying high levels of burnout in all dimensions. Specifically, we confirmed our finding from the pre-pandemic era of very high rates of burnout in terms of personal accomplishments among dialysis unit personnel [18]. This may be related to the specific settings of the medical care provided in dialysis units, which frequently lead to monotony and distress, further intensified by the long-term contact with patients who are often frustrated and depressive [26]. Noteworthy here is that all these burnout-exacerbating pre-existing factors are compounded by a lack of opportunity to thoroughly adjust work in dialysis units amidst the pandemic, which could therefore lead to feelings of increased risk when compared to other healthcare professionals who work in different medical fields and could more readily turn to telemedicine services. Patients had to travel to and from dialysis units as well as share the dialysis hall with others, which made the prevention and control of COVID-19 in dialysis units different from that in the general population and particularly challenging. The demands relating to dialysis therapy varied across the globe—striking inequities were identified in terms of the care of chronic HD patients during the pandemic reported in an ISN/DOPPS (The International Society of Nephrology/Dialysis Outcomes and Practice Patterns Study) survey [27].

The specific character of these healthcare settings forced the adoption of unusual coping strategies from the very beginning of the pandemic, such as ensuring strict infection control, training hemodialysis patients and medical staff, and providing isolated dialysis to close contacts, suspected cases, and confirmed cases of COVID-19 [28]. Another strain on dialysis personnel may have been the stigma from friends and family as they work in such a high-risk area for SARS-CoV-2 contamination [29].

All these circumstances meant that it became crucial to ensure a safe work environment and provide staff with even greater support than before. The results of our study indicate that the work environment at the time of a pandemic is of pivotal significance when dealing with the burnout measures among dialysis healthcare professionals. The most important factors identified were a sense of control over contact with the virus provided by proper support and training, adequacy of personal protection equipment, and sufficient information from the management on current safety procedures. Our results are convergent with data from Romania which showed that practical training sessions on the use of personal protective equipment may reduce emotional exhaustion and increase feelings of mental comfort among medical residents [30]. Also, Swiss investigators found that access to PPE, perceived support by employers, and passage of information from employers are factors responsible for lower burnout and better psychological condition at the time of a pandemic [31].

The same psychometric instruments and comparable study cohorts in our study and the UK national survey of nephrology workforce enabled a comparison of burnout prevalence, which was 14% for depersonalization, 32% for low personal accomplishment, and 41% for emotional exhaustion in the UK cohort, and 24% for depersonalization, 48% for low personal accomplishment, and 39% for emotional exhaustion in our study group. The UK report was not focused specifically on hemodialysis personnel, which may explain the higher rates of participants perceiving low personal accomplishment in our cohort. Selvaskandan et al. found that burnout was more common among younger respondents and those with long COVID [22]. In our survey, we did not address long COVID specifically, but we did not observe associations between burnout and SARS-CoV-2 infections.

Mc Keaveney et al. examined the experiences of renal healthcare practitioners during the COVID-19 outbreak in their mixed-methodology international study, which revealed a high psychological impact of the pandemic, mostly in terms of emotional exhaustion and mental health distress. There was also no specific focus on dialysis personnel in this study, which we assumed to be a proper approach taking into account the above-mentioned set of distinctive factors typical for dialysis care. This study group comprised a vast majority of nurses (86.9%), and only 4.4% of medical practitioners. A high burnout level of emotional exhaustion was perceived by 35.9% of study participants, depersonalization by 16.7%, and low personal accomplishment by 21.1% [21]. Comparing these data with the subgroup of nurses in our study, we observed a higher percentage of nurses perceiving a high level of occupational burnout in the domain of personal achievement. This may be associated with the focus on dialysis personnel in our study. Also, different periods of the pandemic were addressed in both studies—the first lockdown (June 2020–September 2020) in the aforementioned international report, whereas we carried out the survey at the time of the third COVID-19 wave in Poland (September 2021–December 2021). It may be hypothesized that we observed an accumulation of distress and burnout symptoms in the later period of the pandemic.

Effective leadership and supervisor support are cornerstones of a safe and productive work environment, especially in such challenging times. From the perspective of a pandemic, the importance of emotional intelligence consisting of self-awareness, self-management, social awareness, and relationship management is emphasized as a critical aspect of leadership [32]. Our data revealed positive staff evaluation of the assessment, initiatives, and support provided by the leaders. It is of note that these factors were involved in developing a sense of control over contact with the virus, which significantly alleviated burnout in all dimensions. High scores for initiatives and practices by supervisors and leaders in Polish dialysis units indicate their huge dedication and commitment. Hebles et al. underlined the importance of supervisor support for psychological safety within healthcare teams amidst the pandemic [33].

Interventions to reduce burnout are either person-directed or organization-directed. It is argued that taking into account the characteristics of the current crisis with its related time pressure, organization-directed interventions should be prioritized since they may result in faster effects [34]. The framework created in our study on the basis of answers to the open-ended question on issues that could help and relieve staff amidst the pandemic revealed similar expectations with a majority of work-related factors. However, the unprecedented challenge of COVID-19 led to the introduction of non-standard methods in helping address the psychological distress of healthcare professionals. Italian researchers confirmed that in an emergency situation, it is possible to introduce music therapy intervention targeted at staff exposed to stressful events [35].

Among the limitations of our study, low generalizability should be considered. Due to the national character of the survey, the results may be biased by specific local healthcare system-related conditions and COVID-19 restrictions introduced by the government. Another bias that should be taken into account is a social desirability bias which may play a role when reporting one’s feelings in socially sensitive areas like depersonalization-related items in the Maslach Burnout Inventory. Taking into consideration the population of Polish dialysis physicians and nurses as a whole, the group of 363 individuals might be considered a limitation, but the sample size was comparable to the international cohort of 251 participants [21] and 423 respondents in the British survey [22]. In our study we did not collect data on number of all hemodialysis patients in the unit and its localization. Based on the literature, urban hospital physicians had more demanding jobs, less job control and exhaustion caused by burnout, while rural hospital physicians had less social support [36]. In these particular circumstances of the COVID-19 pandemic, social support could play a crucial role and might minimize the harmful effect of burnout among urban doctors. Also, we did not collect specific data on the number of patients with COVID-19. Answers could be biased by lack of knowledge on exact numbers per specific period of time.

### Conclusions

The COVID-19 pandemic has largely affected work in dialysis units. Our results suggest that providing staff with proper training, equipment, and organizational support, which gives a sense of control over the risk of infection, may lead to lower burnout among dialysis nurses and physicians. Leadership and support by the supervisors were positively evaluated by the dialysis staff. Work environment was found to be a crucial factor in alleviating psychological distress amidst the pandemic in this vulnerable group of healthcare professionals. Our results may be useful in the management of future health crises which could affect the well-being and psychological condition of dialysis personnel.

## Data Availability

The datasets generated and/or analyzed during the current study are available from the corresponding author on reasonable request.
